# Effects of electroconvulsive therapy on cerebral A_1_ adenosine receptor availability: a PET study in patients suffering from treatment-resistant major depressive disorder

**DOI:** 10.3389/fpsyt.2023.1228438

**Published:** 2023-07-14

**Authors:** Tina Kroll, Michael Grözinger, Andreas Matusch, David Elmenhorst, Ana Novakovic, Frank Schneider, Andreas Bauer

**Affiliations:** ^1^Institute of Neuroscience and Medicine (INM-2), Forschungszentrum Jülich GmbH, Jülich, Germany; ^2^Department of Psychiatry, Psychotherapy and Psychosomatics, Medical Faculty, RWTH Aachen, Aachen, Germany; ^3^Multimodal Neuroimaging Group, Department of Nuclear Medicine, University Hospital Cologne, Cologne, Germany; ^4^University Hospital, Heinrich Heine University Düsseldorf, Düsseldorf, Germany

**Keywords:** A_1_ adenosine receptor, electroconvulsive therapy, positron emission tomography, major depressive disorder, seizure quality

## Abstract

**Introduction:**

Sleep deprivation and electroconvulsive therapy (ECT) effectively ameliorate symptoms in major depressive disorder (MDD). In rodents, both are associated with an enhancement of cerebral adenosine levels, which in turn likely influence adenosinergic receptor expression. The aim of the current study was to investigate cerebral A_1_ adenosine receptor (A_1_AR) availability in patients with MDD as a potential mediating factor of antidepressant effects of ECT using [^18^F]CPFPX and positron emission tomography (PET).

**Methods:**

Regional A_1_AR availability was determined before and after a series of ECT applications (mean number ± SD 10.4 ± 1.2) in 14 subjects (4 males, mean age 49.5 ± 11.8 years). Clinical outcome, measured by neuropsychological testing, and ECT parameters were correlated with changes in A_1_AR availability.

**Results:**

ECT had a strong antidepressive effect (*p* < 0.01) while on average cerebral A_1_AR availability remained unaltered between pre-and post-ECT conditions (*F* = 0.65, *p* = 0.42, mean difference ± SD 3.93% ± 22.7%). There was no correlation between changes in clinical outcome parameters and regional A_1_AR availability, although individual patients showed striking bidirectional alterations of up to 30–40% in A_1_AR availability after ECT. Solely, for the mean seizure quality index of the applied ECTs a significant association with changes in A_1_AR availability was found (*r*_s_ = −0.6, *p* = 0.02).

**Discussion:**

In the present study, therapeutically effective ECT treatment did not result in coherent changes of A_1_AR availability after a series of ECT treatments. These findings do not exclude a potential role for cerebral A_1_ARs in ECT, but shift attention to rather short-termed and adaptive mechanisms during ECT-related convulsive effects.

## Introduction

Major depressive disorder (MDD) severely influences life quality and is one of the major causes of suicides. Currently, over 320 million people are affected by MDD worldwide (1). The neurobiological understanding of mood disorders has shifted its focus some 20 years ago from monoaminergic transmission to neural plasticity and resilience (2, 3). Besides classical pharma-and psychotherapy, electroconvulsive therapy (ECT) has been established as effective treatment for MDD putatively via increase of adenosine concentrations (4).

Adenosine, acting as a neuromodulator, binds to four subtypes of G-protein-coupled receptors, A_1_, A_2A_, A_2B_ and A_3_. Of these, the inhibitory A_1_ adenosine receptor (A_1_AR) shows highest affinity toward adenosine (5). In the rodent and human brain, A_1_ARs are ubiquitously expressed (6, 7) in neurons on somato-dendritic and axonal pre-and post-synaptic sites as well as in glia cells. Activation of presynaptic A_1_ARs mainly restrains the release of excitatory transmitters like glutamate from synaptic terminals, while the postsynaptic activation ultimately hyperpolarizes the membrane and inhibits signal transduction (8). With these mechanisms adenosine exerts homeostatic control of brain function, e.g., in epileptogenesis (9) and sleep–wake cycle (10), regulates synaptic plasticity, and supports neuroprotection in ischemic, hypoxic and oxidative stress events (11, 12).

Adenosine as the nucleoside precursor of the adenine-nucleotides AMP, ADP, and ATP stands in dynamic equilibrium with these compounds and is crucial for the energy metabolism of living organisms. In the extracellular space of the brain, adenosine either derives from the metabolism of adenosine-triphosphate (ATP) or is released from cells via bidirectional nucleoside transporters (5). Therefore, its concentration is closely related to energy consumption and sensitively indicates the balance between energy demand and supply on the synaptic level.

Specifically, adenosine has been identified as an endogenous anticonvulsant (9, 13). The high ATP breakdown during generalized seizures generates an excessive amount of adenosine with a transient increase of extracellular adenosine concentrations of more than 20-fold. In turn, consecutive activation of inhibitory A_1_ARs induces seizure arrest and postictal refractoriness (14). However, data on chronic regulation of A_1_AR in epilepsy remains inconclusive with observed up-and downregulations of the receptor in humans (15, 16) and rodent models (17–19). But, series of ECS in rats, artificially inducing seizures and mimicking human ECT, led to an increased expression of A_1_ARs in the cortex and other regions of the rat brain (20, 21).

In humans, therapeutic initiation of seizures with a subsequent surge of adenosine in terms of ECT is a potent therapeutic option in either severe or medication resistant depression (22–24). The effect of ECT seems to be rather associated with the intensity of generalized seizures than with the electric current (25). However, the molecular mechanisms of the antidepressant effects are not completely understood. Enhanced A_1_AR signaling either via transgenic knock-in or via injection of an A_1_AR agonist in wild-type mice was associated with a distinct resilience toward depressive-like behavior, whereas A_1_AR knockout induced opposite effects (26). Thus, adenosine and an upregulation of its A_1_AR might be involved in the persistent antidepressant efficacy of ECT particularly after repeated applications. Moreover, A_1_AR knockout mice showed a resistance toward antidepressant effects of SD, another well-established therapeutic option of MDD (27).

Altogether, there is evidence that adenosine and central inhibitory A_1_ARs might be crucial for antidepressant efficacy of primary non-pharmacological therapeutic interventions like ECT in MDD. As there is an urgent need of fast and reliably acting antidepressant therapies, especially in suicidal patients, it is essential to understand the molecular base of non-medical treatment options to refine therapeutic interventions.

The main aim of the current study was to investigate cerebral A_1_AR availability as a potential neurochemical basis for sustained ECT effects in patients with medication resistant severe MDD as originally proposed by van Calkar and Biber (28). A_1_AR availability was determined by the A_1_AR antagonist 8-cyclopentyl-3-(3-[^18^F]fluoropropyl)-1-propylxanthine ([^18^F]CPFPX) and positron emission tomography (PET) (29–31) before and after a series of ECT applications. As a secondary outcome, results were additionally correlated to clinical ratings of therapeutic effects, cognitive side effects as well as to inherent parameters of ECT like stimulus charge and seizure quality.

## Material and methods

### Subjects and study design

All study participants were inpatients of the Department of Psychiatry, Psychotherapy and Psychosomatics of the University Hospital, Aachen, Germany. Fifteen subjects (mean age 49.5 ± 11.8 years, range 26.2–63.9 years, 11 females) were included in the study after obtaining written informed consent. Except for one female, all subjects were right-handed. One of the females was excluded during data analysis due to cortical atrophy. For demographic parameters of the remaining 14 subjects, see Table 1.

**Table 1:** Demographic and illness-related data as well as ECT treatment and quality parameters of study subjects.

	Min	Max	Mean ± SD
Demographic parameters^#^			
Age [years]	26	64	49.5 ± 11.8
BMI [kg/m^2^]	19	38	26.5 ± 5.5
Verbal IQ [vocabulary test]	83	122	103.9 ± 11.8
Sleep duration [hrs/night]^1,2^	5.3	13.5	7.9 ± 3.5
Caffeine [dl/d; *n* = 12]^1,3^	1.5	10.3	3.5 ± 2.6
Nicotine [cigarettes/d; *n* = 11]^1,3^	4	30	13 ± 11
Illness-related parameters			
Age at illness onset [years]	13	63	32.4 ± 15.4
Age at first hospitalization [years]	17	63	38.8 ± 16
Duration of illness [months]	9	464	203.7 ± 138.4
Number of depressive episodes	1	6	3.7 ± 1.5
Number of hospitalizations	1	10	3.9 ± 2.2
Number of suicide attempts	0	3	0.79 ± 0.89
Duration of current episode [months]	1	24	9.4 ± 7.5
ECT treatment parameters			
Number of ECT sessions	8	12	10.4 ± 1.2
Stimulus charge [mC]^4^	218	719	415 ± 138.2
Seizure duration [seconds]^4^	45	106	69.4 ± 19.8
Stimulus charge of last treatment [mC]	227	1,008	520.2 ± 207.7
ECT quality parameters			
Maximum sustained power [μV]^4^	4,905	30,805	13798.2 ± 7844.8
Maximum sustained coherence [%]^4^	83	97	88.9 ± 4.5
Peak heart rate [bpm]^4^	107	159	130.6 ± 14.3
Postictal suppression^4,5^	0.2	0.88	0.5 ± 0.2
Quality rating of ECT sessions [%]^4^	51	96	82 ± 11

*n* = 14, ^#^data given for the time-point of the baseline PET scan, ^1^self-reported via questionnaire, ^2^nearly all subjects reported different kinds of sleep disturbances, ^3^patients without caffeine (*n* = 2) and non-smokers (*n* = 3) were not included in the average, ^4^average across treatments for each subject, ^5^visually scored: 1 for sufficient, 0 for insufficient postictal suppression; BMI, body mass index; ECT, electroconvulsive therapy; IQ, intelligence quotient; SD, standard deviation.

Subjects had been diagnosed with recurrent moderate or severe major depressive episodes according to ICD-10 criteria (German edition, F33.1 (*n* = 1), F33.2 (*n* = 9) that were accompanied by psychotic symptoms (F33.3) in three subjects). One subject suffered from a single severe depressive episode with psychotic symptoms (F32.3). Psychiatric comorbidities were present in two subjects (personality disorder (F61, one male) and somatic symptom disorder (F45.0, one female)). All participants were classified as medication-resistant and had undergone at least two adequate antidepressant medications with different mechanisms of action. For further illness-related parameters, see Table 1.

At the time of study investigations all subjects were free of comorbidities or medication with a known influence on adenosine receptor expression. Further information on given medication can be found in Supplementary material and methods; Supplementary Table S1.

Subjects underwent baseline neuropsychological examination (4.1 ± 2.4 days before the PET investigation) and subsequently received the first baseline (BL) PET scan at the PET laboratory of the Institute of Neuroscience and Medicine, Forschungszentrum Jülich. ECT treatments started after a time interval of 1.36 ± 0.84 days after BL PET. After 10.4 ± 1.2 (range 8–12) ECT sessions, the second PET scan (follow-up, mean time lag between BL and follow-up PET: 43 ± 6.4 days) was scheduled with a time lag of 5.71 ± 2.7 (range 2–13) days after the last ECT session to minimize interference of acute effects with more persistent alterations of A_1_AR availability. Further neuropsychological ratings were scheduled 2.5 ± 4 days after the second PET scan. Prior to both PET scans, subjects refrained from any caffeine consumption for at least 36 h to preclude influences on PET pharmacology (32). All procedures were carried out on accordance with the Declaration of Helsinki and were approved by the Ethics Committees of the University Hospitals Düsseldorf and Aachen as well as by the German Federal Office for Radiation Protection.

### Neuropsychological ratings

Neuropsychological test batteries for both ratings consisted of Beck Depression Inventory-2 (BDI-2), Global Assessment of Functioning (GAF), Montgomery-Åsberg Depression Rating Scale (MADRS), and Hamilton Depression Scale (HAMD-21). Mini Mental State Examination (MMSE), Verbal Learning and Memory Test (a modified version of Rey Auditory Verbal Learning Test, RAVLT), Wechsler Memory Scale (Working Memory (WMS WM) and Short Term Memory (WMS STM)) were supposed to measure potential cognitive side effects of ECT treatment. Intelligence Quotient (IQ) was estimated by *Wortschatztest* (WST), a German test for passive vocabulary. All ratings were conducted by psychologists of the neuropsychological team of the Department of Psychiatry, Psychotherapy and Psychosomatics of the University Hospital Aachen.

### ECT treatments

Treatments were started with right unilateral (RUL) electrode position, a pulse width of 0.5 ms and a charge according to the age of the patient (33). Etomidate was applied as a narcotic (average dose 24.4 ± 5.6 mg) and succinylcholine as a muscle relaxant (average dose 113.7 ± 19.1 mg). Due to a strong anticonvulsive reaction, the narcotic was changed to S-ketamine (average dose 91.7 ± 28.9 mg) in three subjects during the course of the treatments.

The regular treatment frequency was twice a week with occasional reduction to one session in case of strong short-term cognitive side effects or required cardiovascular diagnostic assessments. In five patients, the electrode position was changed from RUL to left-anterior-right-temporal (LART) after at least six treatments due to unsatisfying clinical improvement. Re-Stimulation was performed in 1 to 4 ECT sessions of five patients with insufficient seizures. Further details on ECT treatment parameters are given in Table 1. All treatments were performed with the Thymatron System IV (SOMATICS, INC, Lake Bluff, IL, USA) in accordance with routinely used procedures (34–37).

Subsequent to the treatment, seizure quality was rated by analysis of the two frontal EEG and the ECG recordings according to the following criteria: EEG seizure duration >20 s, midictal amplitude >180 μV, maximal sustained coherence >85%, maximum heart rate > 110/min, and level of postictal suppression (see Table 1). Since the automatic evaluation of the postictal suppression index was often distorted by technical problems, it was qualitatively assigned to the categories ‘sufficient, insufficient or not applicable’ based on visual inspection of the investigating physician. All other parameters were based on the output of the Thymatron System. Seizure quality index for each session was given as the percentage of all applicable and fulfilled criteria ranging from zero to 100%. To compensate for the anticonvulsive effect, the stimulus charge was increased by 100 mC when the seizure quality turned out low.

### Imaging procedures and data analysis

PET imaging was performed in supine position in quite ambiance with an ECAT EXACT HR+ Scanner (Siemens CTI). Scan duration was 90 min and started simultaneously with radiotracer injection via bolus-infusion with a *K*_bol_ of 63 min. [^18^F]CPFPX was produced in-house as previously described (31). Arterialized venous blood samples were collected 1, 5, and 10 min after scan start with subsequent sampling every 10 min.

Determination of radioactivity in whole blood and plasma as well as plasma metabolite analyses were performed as described previously (38). A_1_AR availability was determined as the tissue-to-plasma ratio (TPR) under equilibrium conditions from minutes 50–90. Under these conditions, the TPR equals the total distribution volume (*V*_T_) and can be expressed as the ratio of activity concentration in the respective region of interest (*C*_ROI_) and the metabolite-corrected plasma (*C*_P_) input function: *V*_T_ = *C*_ROI_/*C*_P_. Further information on image processing is given in the Supplementary material and methods.

### Statistical analyses

All data was first analyzed with regard to normal distribution via Shapiro–Wilk test. Differences in general parameters of PET scans as well as therapeutic impact of ECT and its potential side effects, determined via neuropsychological testing, were analyzed by two-tailed paired t-tests of the baseline and follow-up parameters. Interferences of regional A_1_AR availability by ECT treatment were further investigated with linear mixed model analysis with brain region and time as fixed parameters and random intercepts attributed to patients.

To examine relationships of an A_1_AR related molecular basis and antidepressive effects of ECT treatment, the average differences in A_1_AR availability across brain regions of each patient were correlated with individual neuropsychological rating scale changes. The same procedure was applied to determine influences of study design related parameters, anticonvulsive effects of ECT treatment, individual case history, and ECT quality indexes on changes in distinct A_1_AR availability. Pearson’s correlation coefficient (*r*) or Spearman’s rho (*r*_s_) were determined in dependency of normal distribution of underlying data. Level of significance was set to α < 0.05. All calculations were performed using SPSS Statistics (version 24.0, IBM Corp., Armonk, NY, USA).

## Results

Baseline and follow-up PET scans were not significantly different regarding daytime of scan, injected dose, molar activity at time of injection, injected amount of substance, and mean rate of change of parent compound in plasma as an indicator for quality of equilibrium (see Supplementary Table S2).

Subject-specific parameters like BMI, sleep duration per night, caffeine intake and nicotine consumption did not differ between both scans. In some subjects, medication had to be modified due to patients complaining about side effects or to improve seizure quality.

**Figure 1:**
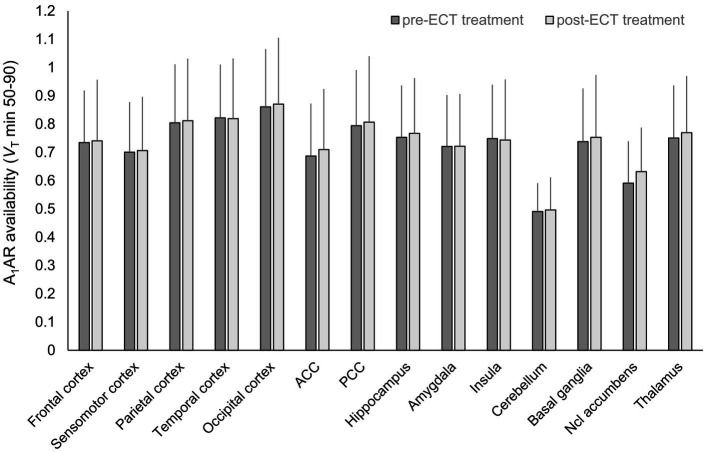
Regional A_1_AR availability before and after ECT treatment. *n* = 14, mean ± standard deviation, A_1_AR, A_1_ adenosine receptor; ACC, anterior cingulate cortex; ECT, electroconvulsive therapy; Ncl, nucleus; PCC, posterior cingulate cortex; *V*_T_, distribution volume in tissue.

**Figure 2:**
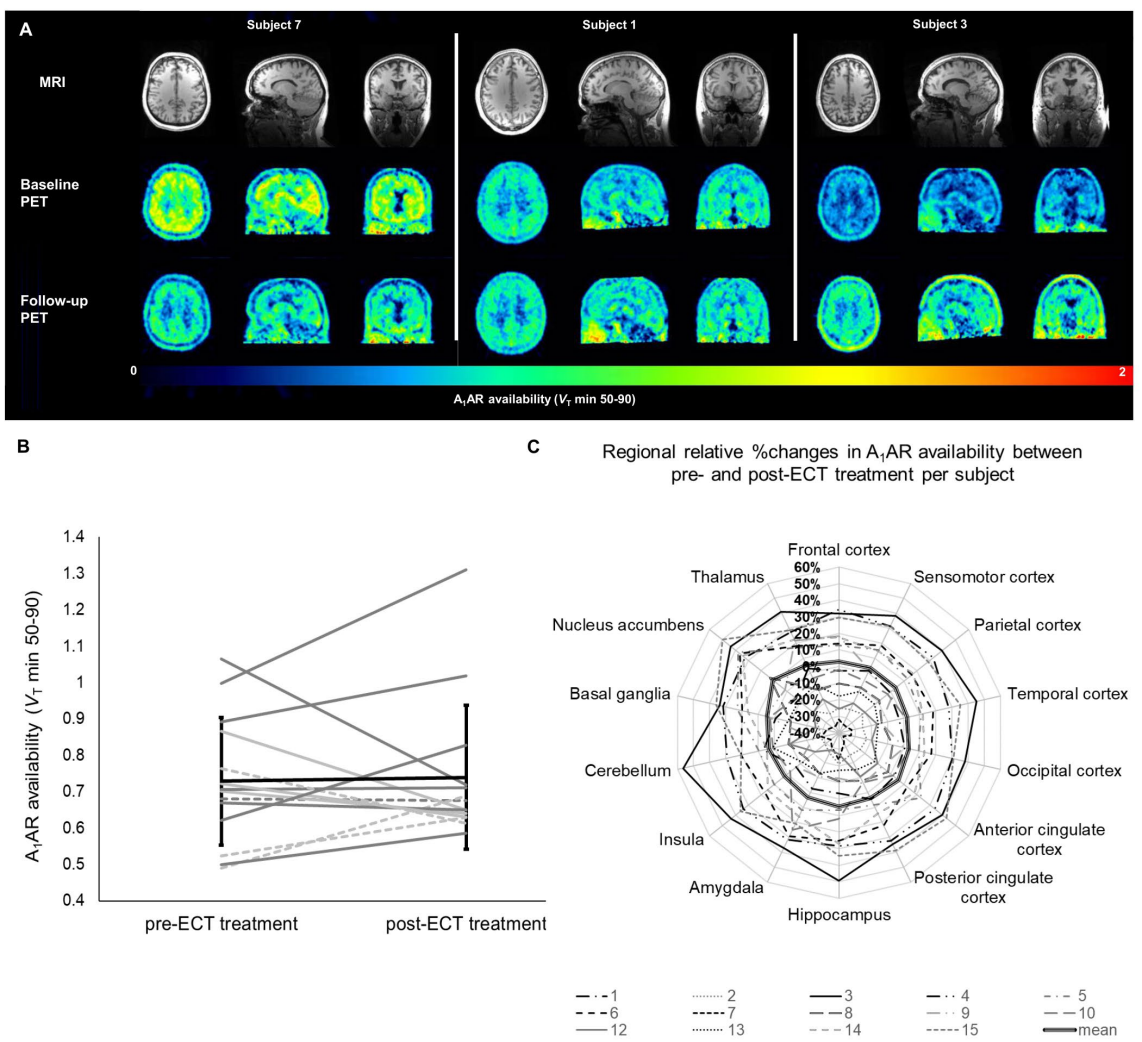
Individual changes in A_1_AR availability in relation to ECT treatment. **(A)** Parametric PET images demonstrating A_1_AR availability of three subjects representing the highly individual A_1_AR responses to ECT treatment. Given are transversal (left), sagittal (middle), and coronal (right) planes. Upper row shows individual MRI images used as anatomical references. **(B)** Mean A_1_AR availability per subject averaged over all investigated regions. Each line represents one subject, dotted lines depict male subjects, patients diagnosed other than F33.2 (including psychiatric comorbidities) are marked in light gray. Mean ± standard deviation for all subjects is given in black. **(C)** Regional relative changes given in % of pre-ECT A_1_AR availability. Each line depicts one subject. Bold line represents the mean of all subjects (*n* = 14). A_1_AR, A_1_ adenosine receptor availability; ECT, electroconvulsive therapy; MRI, magnetic resonance imaging; PET, positron emission tomography; *V*_T_, distribution volume in tissue.

Treatment of subjects with ECT resulted in a strong decline of the depressive symptoms as shown in Table 2 (upper part). None of the cognitive tests yielded significant differences before and after ECT treatment (Table 2, lower part). Regional A_1_AR availability as measured before and after ECT treatments is shown in Figure 1 (for absolute values see Supplementary Table S3). Distribution of cerebral A_1_ARs is heterogeneous as depicted in representative PET images of individual subjects for both the baseline as well as the follow-up condition (see Figure 2A).

**Table 2:** Scores of neuropsychological assessments before and after ECT treatments.

Rating	Baseline	Follow-up	*P-*value (paired *t*-test)	Effect size (Cohen’s *d*)
HAMD-21	26.29 ± 5.80	11.93 ± 5.01	**<0.001**	2.04
BDI-2	37.07 ± 9.49	14.43 ± 8.38	**<0.001**	1.80
GAF	49.71 ± 4.12	62.64 ± 5.94	**<0.001**	−2.35
MADRS	29.43 ± 5.53	11.07 ± 5.58	**<0.001**	2.85
MMSE	29.57 ± 1.09	29.00 ± 0.88	0.071	0.52
VLMT	49.43 ± 13.05	52.57 ± 14.25	0.323	−0.27
WMS (dsf)	8.57 ± 2.10	8.14 ± 1.92	0.396	0.23
WMS (dsb)	7.36 ± 2.02	7.29 ± 2.43	0.818	0.06
WMS (stm)	60.21 ± 32.80	54.50 ± 30.78	0.467	0.20
WMS (wm)	57.29 ± 31.69	59.43 ± 34.38	0.575	−0.15

Values reported as mean ± standard deviation, *n* = 14, BDI-2, Beck’s Depression Inventory; GAF, Global Assessment of Functioning; HAMD-21, Hamilton Depression Rating Scale; MADRS, Montgomery-Åsberg Depression Rating Scale; MMSE, Mini Mental State Examination; VLMT, Verbal Learning and Memory Test; WMS, Wechsler Memory Scale; dsf, digital span forward; dsb, digital span backward; stm, short-term memory; wm, working memory. Significant differences are marked in bold.

In mixed model analysis the variable brain region significantly influenced A_1_AR availability (*F* = 12.8, *p* < 0.01), but not the variable time-point of measurement (*F* = 0.65, *p* = 0.42). As shown in Figure 2B alterations in A_1_AR availability across regions between pre-and post-ECT at group levels were marginal (mean ± SD average difference 3.93% ± 22.7%). Mean relative difference in A_1_AR availability per region (see Figure 2C; Supplementary Table S3) ranged from 2.03% ± 24.5% (insula) to 10.93% ± 27.85% (nucleus accumbens). Moreover, there was no interaction between brain region and time-point of measurement (*F* = 0.06, *p* = 1).

Noticeably, at the individual level subjects showed highly variable responses ranging from inclines of A_1_AR availability across regions after ECT treatment of 40% to declines of 32% (see Figure 2B). Nevertheless, at the level of specific brain regions, each subject showed high coherence in A_1_AR response over all investigated regions after ECT treatment (see Figure 2C) with a low within-subject share related to the 14 investigated brain regions of around 8% of the total variance of the differences in A_1_AR availability. According to the small contribution of the variable brain region to total variance, for further analysis the relative differences in A_1_AR availability between baseline and follow-up PET were averaged across all brain regions for each patient and then correlated with the individual clinical outcome determined via repeated neuropsychological ratings. Neither a relationship between A_1_AR variations and clinical responses nor a link to potential cognitive side effects of ECT treatment were found (see Supplementary Table S4). In line with this, an exploratory linear mixed model analysis with inclusion of the fixed factor response to ECT treatment (defined as a reduction of HAM-D scores of more than 50%) revealed no influence of clinical response on A_1_AR availability in relation to ECT treatment (*F* = 0.51, *p* = 0.49).

**Figure 3:**
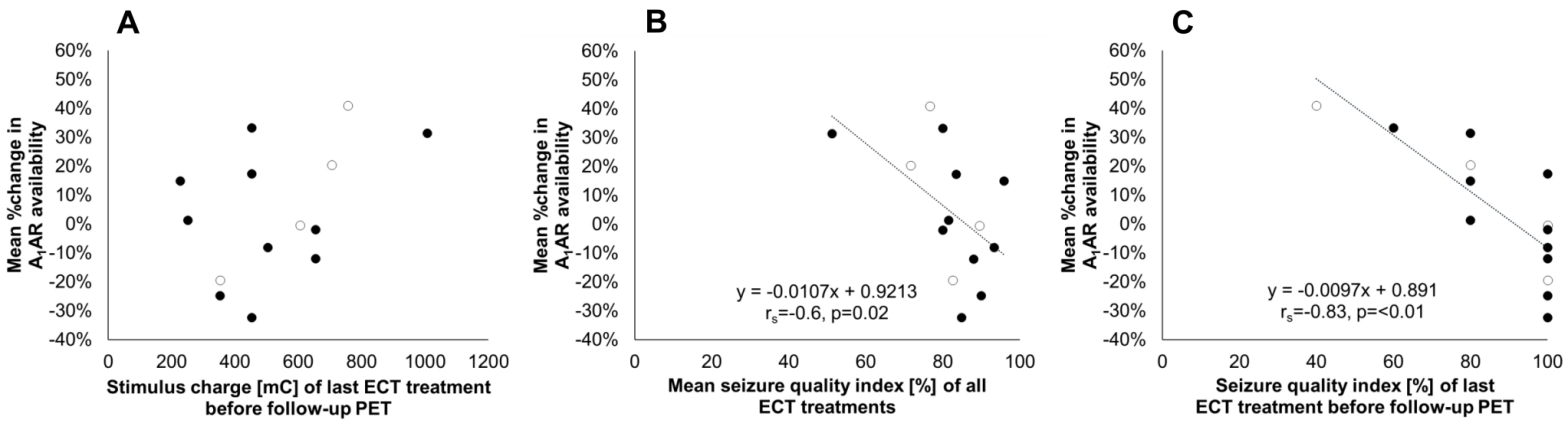
Changes in A_1_AR availability between pre-and post-ECT treatment in relation to treatment parameters. The vertical axis indicates the relative change in A_1_AR availability averaged across investigated regions per subject related to the respective baseline value before treatment. The horizontal axis indicates **(A)** Stimulus charge of the last ECT treatment before follow-up PET. **(B)** Seizure quality index as a mean of all ECT treatments. **(C)** Seizure quality index of the last ECT treatment before follow-up PET. Regression line and parameters for significant correlations are depicted in the graphs **(B,C)**. Open circles represent male subjects. *n* = 14. A_1_AR, A_1_ adenosine receptor; ECT, electroconvulsive therapy; PET, positron emission tomography; *r*_s_, Spearman’s rho.

Furthermore, neither parameters related to study design like the number of applied ECT treatments (*r* = 0.35, *p* = 0.22) nor the time interval between the last ECT treatment and the follow-up PET (*r*_s_ = −0.18, *p* = 0.54) nor individual case history with regard to total duration of depression (*r* = −0.17, *p* = 0.57) and number of depressive episodes (*r* = −0.42, *p* = 0.13) influenced A_1_AR responses in the investigated subjects. Stimulus charges of the last ECT prior to the follow-up PET (*r* = 0.44, *p* = 0.12, see Figure 3A) as well as the relative difference in stimulus intensities related to the initial charge of the first treatment session (*r* = 0.12, *p* = 0.7) as indicators of the anticonvulsive impact of ECT did not reveal any significant links to individual A_1_AR changes either.

However, for the mean seizure quality index of the applied ECT treatments a significant association with average differences in A_1_AR availability across investigated regions per subject was found (*r*_s_ = −0.6, *p* = 0.02, see Figure 3B), linking strong increases of receptor availability to low stimulus quality. This link was even more pronounced when correlating changes in A_1_AR availability with the seizure quality index of the last ECT treatment before follow-up PET (*r*_s_ = −0.83, *p* = <0.01, see Figure 3C). Explorative analysis revealed that the maximal sustained coherence as part of the seizure quality index seems to be the main responsible parameter for the observed relationship.

## Discussion

The present longitudinal *in vivo* study is to the best of our knowledge the first examining the relationship of cerebral A_1_AR availability, ECT treatment and clinical outcome parameters in medication-resistant depressive patients. Our main findings reveal that A_1_AR availability did not differ before and 5.7 days (2–11 days) after ECT treatment in any of the evaluated brain regions on the group level of investigated subjects. Meanwhile ECT proved to be clinically effective in all subjects with a considerable decrease of depressive symptoms. Although no group effects were detected, courses of A_1_AR availability were highly variable across patients, with inclines and declines of up to 30–40%. Interestingly, individual changes in A_1_AR availability were highly concordant across all investigated regions which points to stable individual reactions to ECT. However, these did not correspond to individual clinical outcomes and most treatment features. Solely mean seizure quality indexes showed correlations with individual changes in A_1_AR availability over time, a relationship which was even stronger when only the last ECT treatment before the follow-up PET was considered.

Previous preclinical studies indicated significant effects of adenosine (39) and the cerebral A_1_AR (26) on depressive behavior. In naïve mice, Kaster et al. (39) demonstrated effects of intraperitoneal adenosine injections on immobility in the forced swim as well as tail suspension test, being widely considered as a sign for depressive-like behavior. Serchov et al. (26) showed in the chronic behavioral despair mouse-model that increasing transgenic A_1_AR expression ameliorated typical depressive-like behaviors in paradigms like the forced swim test, tail suspension test and sucrose preference test. Given these observations as well as the fact that ECS resulted in an increased expression of A_1_ARs in rodents (20, 21), it was rather unexpected that on average A_1_AR availability in humans remains constant after a series of ECT applications. However, respective effects were observed in animal models and it is an ongoing debate to what extend rodent models comprehensively resemble the complex human condition of MDD. Current models of depression should meet criteria such as face validity (similarity of symptoms), predictive validity and construct validity and it is unambiguously hard to create models achieving high scores in all of these parameters (40). Especially it is at least challenging to separate the pathologies of chronic stress and depression in animal models. Thus, although indicators of behavioral despair in chronic unpredictable stress models rated high in specificity of depressive-like symptoms (41) it has to be critically reflected if repeated chronic swim stress as a rather severe stressor in mice might predominantly provoke stress responses at behavioral and molecular levels. In this context, Crema et al. (42) revealed an upregulation of the A_1_AR in the chronic unpredictable mild stress model and the chronic restraint stress model, both being used as preclinical models of depression. However, only animals in the chronic unpredictable mild stress group showed depressive-like behavior indicating that the observed regulation of the A_1_AR was primarily triggered by chronic stress. Behavioral readouts in rodent models of depression are critically discussed in the literature because of primarily assessing immobility, e.g., in the forced swim and tail suspension test, and thus coping with stressful situations rather than depression-like behavior (40, 43). Complexity of symptoms in human depression and related difficulties to model and translate subjective human emotions to specific rodent behaviors should be taken into account when interpreting and translating preclinical data to the clinical situation.

In humans, therapeutic ECT is performed in a conservative manner including muscle relaxation, carefully selected stimulation paradigms with limited total charge, continuous monitoring of the vital signs and a treatment frequency of 2–3 per week to increase tolerability. In particular, especially the hyper-oxygenation prior to the treatment and the maintenance of a constant oxygen saturation of 100% might be a crucial difference between human ECT regimes and rodent ECS studies for which no such precautions are described. In a cell model of smooth muscle cells chronic hypoxia thus led to a 3.5 fold increase in maximum specific binding of the A_1_AR antagonist [^3^H]DPCPX (44). Moreover, under transient hypoxic conditions, cultured astrocytes react with increasing extracellular adenosine levels to maintain brain homeostasis via reduction of presynaptic excitatory transmitter release in an A_1_AR-dependant mechanism which ultimately downregulates synaptic activity during seizures (45). In summary, preclinical investigations on ECS cannot be directly compared with the human condition – neither with regard to the strength of the seizure and the consecutive level of adenosine nor in view of other physiological parameters having an potential impact on energy metabolism and adenosine signaling. These discrepancies might result in longer-lasting and more intense regulations of the A_1_AR in rodent models due to stronger stimuli.

Altogether, the previously observed increases in A_1_ARs are more likely an adaptive mechanism than reflecting the molecular base of the long-lasting antidepressant efficacy of ECS/ECT. Indeed, adenosine and the A_1_AR act in an anticonvulsive manner ultimately terminating pathological seizures [for review see (46)]. Adenosine surges and an altered A_1_AR expression might cause insufficient therapeutic seizure generation, short seizure duration as a component of seizure quality, as well as the need to adapt the stimulus charge during the course of ECTs. Diminished A_1_AR availability post-ECT in subjects with higher seizure quality may thus reflect limited compensatory mechanisms in response to induced convulsions in these subjects. Such A_1_AR mediated coping mechanisms were already observed by Elmenhorst et al. (47) in the context of SD and alcohol intake with subjects showing more pronounced increases in A_1_AR availability after intervention performed better in the psychomotor vigilance task. The dynamic reserve of the A_1_AR, i.e., the potential to increase A_1_AR availability upon stimulus, may thus reflect resilience to convulsions, alcohol and SD. Interestingly, some subjects showed a downregulation of A_1_AR availability after ECT treatment in the current study. This observation is in line with previously observed inconclusive results on regulation of A_1_AR availability in epilepsy as some studies indicated a decrease in receptor availability in both human disease and rodent epilepsy models (16–19). However, reasons for this putative individual vulnerability to convulsions remain unclear and warrants further investigation.

Although no consistent long-term effects of ECT on A_1_AR availability were determined, a more uniform, transient upregulation of the receptor after ECT cannot be excluded as the follow-up PET scan was performed with an average time lag of approximately 6 days after the last ECT treatment in the present study, thus avoiding interferences of acute ECT effects and A_1_AR quantification. In previous preclinical investigations on A_1_AR availability after ECS (20, 21) short-term effects in the immediate postictal phase might have been mimicked by a competition of massively increased endogenous adenosine and the utilized agonistic radioligand.

SD, as another antidepressant therapy induces such short-term upregulations of the A_1_AR (48) but just a single night of recovery sleep after 52 h of SD was able to completely restore A_1_AR availability to baseline levels (49). In line, recovery sleep in patients with MDD lead to a relapse of depressive symptoms in most SD-responders (50). Although SD elicits short-term antidepressive actions and the underlying molecular mechanisms are not completely understood it seems to be at least partly mediated by the A_1_AR (51). Despite the putative short-term antidepressive efficacy, these studies indicate that the turnover of components of the adenosinergic system is at least faster than 2–11 days in humans. This finding supports the hypothesis of a rapid adaption of this system in response to ECT induced adenosine surges but with only short-term antidepressive effects.

A theoretical study design to address short-term interactions of adenosine and the A_1_AR would employ an agonistic radiotracer and continuous PET acquisition during a running ECT treatment. By this, effects of repetitive ECT on adenosine and its binding to A_1_ARs could be investigated. Moreover, as high adenosine surges occurred during ECT and several hints point toward an involvement of the purinergic system in mood disorders, other targets like the adenosine A_2A_ adenosine receptor (A_2A_AR), the ATP receptor P2X7 as well as interactions of the A_1_AR/A_2A_AR and the adenosinergic and dopaminergic system warrant further investigations. Recently, it was shown, that ketamin-induced increases in axon terminal density, measured via determination of synaptic vesicle protein 2 and PET, might be crucial for reduction of depressive symptoms in subgroups of depressed patients with specific molecular characteristics (52). Keeping this in mind, future research on pathophysiological mechanism of MDD should also focus on interactions of the neuromodulator adenosine and restoration of synaptic connections. Furthermore, larger and more homogenous samples would offer the opportunity to classify patients in subgroups on the basis of their molecular and behavioral traits to further elucidate mechanisms of treatment response with the ultimate goal of a more individualized therapy of MDD.

Our rather heterogeneous sample closely depicts the common clinical situation of therapy-resistant severely depressed patients being treated with ECT. However, besides age, heterogeneous treatment parameters as well as individual history of disease, medication and psychiatric diagnosis will surely introduce a relevant variability. In the present sample the coefficient of variation averaged over all patients and investigated regions amounted to 24% before and 27% after the treatment. This is only slightly higher than in our previous studies on effects of SD in which the coefficient of variation was approximately 20% (averaged across brain regions and subjects (48)). Though, taken into consideration that the current sample consists of severely depressed patients of different gender, age, smoker status and medication, interindividual variance of data seems proportional in comparison to the male, young, non-smoking and non-medicated group of healthy volunteers measured in our previous SD studies. Apart from one subject, all patients showed remarkable improvement of symptoms after ECT treatment. Response rates between 50 and 86% (depending on the applied neuropsychological test and cut off) in the current sample are in the expected range (53), further highlighting that the sample is representative despite the relatively small sample size which is however in the range of other clinical PET studies performed in severely diseased patients (54–58). In such investigations especially with observations over longer time periods, oftentimes, individual medication and therapy regimes had to be adapted to the actual status and burden of the patient which automatically resulted in slightly varying treatments over the course of several weeks. Notwithstanding, in the present sample neither individual history of disease nor specific parameters of ECT treatments, like, e.g., number of treatment sessions, time intervals between the last ECT treatment and follow-up PET investigation, electrode position, and changes in stimulus charges had an influence on individual changes of A_1_AR availability. Moreover, usage of S-ketamine in three subjects for anesthesia during ECT did not influence antidepressant effects of ECT treatment in the current sample as relative changes in, e.g., HAM-D in two of these subjects were less than the average change across the sample. In addition, a direct effect of S-ketamine on A_1_AR availability is not known up to now which is mirrored in the current sample with heterogeneous responses of A_1_AR availability after ECT treatment in the respective patients. Nevertheless, one of the main limitations of the current study is the missing control group. However, longitudinal PET imaging with [^18^F]CPFPX was proven reliable with a moderately high test–retest stability as determined on consecutive days (30). Comparisons of test–retest parameters in rodents for different interscan intervals (59, 60) moreover indicates that A_1_AR receptor availability as determined via [^18^F]CPFPX-PET is stable over periods of up to more than 3 months which is longer than the interscan intervals of the current study. Intraindividual changes of A_1_AR availability between baseline and follow-up PET observed in the current study were of greater magnitude as previously determined in the human test–retest study (30). Thus, percentage A_1_AR variability across regions between the two scans of the current study were approximately elevated by half in comparison to standard test–retest conditions (~19% vs. ~13%). Furthermore, coefficients of variation for determined A_1_AR variability between both time points across regions and subjects were additionally increased within the same range (~63% vs. ~40%). In the context of the rather marginal increase of absolute variability in A_1_AR distribution volume measured in patients with MDD in comparison to healthy subjects (see above) and further PET-specific methodological considerations (see Supplementary Discussion), it seems likely, that more patient-centered factors mainly determine the high variability of A_1_AR responses to ECT treatment. Although these factors are not known in detail yet, it appears as they mirror more robust individual characteristics presumably in form of an anticonvulsive response mechanism.

In conclusion, our present study shows that the cerebral A_1_AR does not exhibit long-term changes that could directly explain the antidepressant effects of ECT. Short-term antidepressive efficacy of A_1_AR regulation can, however, not be excluded based on the current study design. Highly individual A_1_AR responses to ECT treatment showed coherence across brain regions within the same subjects but no correlation with treatment outcomes. Future studies on effects of ECT treatment warrant bigger sample sizes to further elucidate the complex interplay of molecular and behavioral changes with putative individual trademarks in the depressive brain.

## Data availability statement

The raw data supporting the conclusions of this article will be made available by the authors, without undue reservation.

## Ethics statement

The studies involving human participants were reviewed and approved by Ethics Committees of the University Hospitals Düsseldorf and Aachen. The patients/participants provided their written informed consent to participate in this study.

## Author contributions

TK, MG, and AB conceptualized and designed the study. MG recruited patients. TK, MG, AM, and AN acquired the data, while TK, MG, DE, and AB analyzed and interpreted data. TK provided a first draft of the manuscript. MG, AM, DE, AN, FS, and AB subsequently revised the manuscript. All authors contributed to the article and approved the submitted version.

## Conflict of interest

The authors declare that the research was conducted in the absence of any commercial or financial relationships that could be construed as a potential conflict of interest.

## Publisher’s note

All claims expressed in this article are solely those of the authors and do not necessarily represent those of their affiliated organizations, or those of the publisher, the editors and the reviewers. Any product that may be evaluated in this article, or claim that may be made by its manufacturer, is not guaranteed or endorsed by the publisher.
