# Supplementary material for: Effects of electroconvulsive therapy on cerebral A1 adenosine receptor availability: a PET study in patients suffering from treatment-resistant major depressive disorder
Source: Front Psychiatry. 2023 Jul 14;14:1228438. doi: 10.3389/fpsyt.2023.1228438 (PMC10380952; doi:10.3389/fpsyt.2023.1228438)
Supplement: Supplementary file 1 [file Data_Sheet_1.PDF]

Supplementary Material

Effects of electroconvulsive therapy on cerebral A_1 adenosine receptor availability:

A PET study in patients suffering from treatment-resistant major depressive disorder

Tina Kroll[†*], Michael Grözinger[†], Andreas Matusch, David Elmenhorst, Ana Novakovic, Frank Schneider, Andreas Bauer

Tina Kroll[†*], Michael Grözinger[†]
[†]These authors contributed equally to this work.

*** Correspondence:**
Tina Kroll
t.kroll@fz-juelich.de

Material and Methods

Psychiatric medication

Thirteen participants received between one and three antidepressants. Lorazepam was given in seven patients with a mean of 1.2 mg/day, the others had no benzodiazepines. Besides antidepressants some subjects were provided with Risperidone (one subject), Quetiapine (two subjects), low-potency antipsychotics (six subjects), and anxiolytics (one subject). Lithium was augmentatively administered in five participants. Whenever the clinical situation allowed, medication was maintained stable. Complete medication of subjects for both PET scans is given in Supplementary Table 1.

Supplementary Table 1 Medication of subjects at timepoint of both PET scans

Subject	Baseline		Follow-up	
	Medication	Dosage	Medication	Dosage
1	Seroquel retard	350 mg	Seroquel retard	350 mg
	Seroquel	50 mg		
	Trevilor retard	225 mg	Trevilor retard	225 mg
			Unizink	20 mg
2	Tianeurax	37.5 mg	Tianeurax	25 mg
	Mirtazapin	7.5 mg	Mirtazapin	7.5 mg
	Quilonum retard	18.3 mmol	Quilonum retard	6.1 mmol
			Pantoprazol	40 mg
3	Delix	5 mg	Delix	5 mg
	Movicol	2 sachets	Movicol	2 sachets
	Mirtazapin	15 mg	Mirtazapin	15 mg
	Quilonum retard	30.5 mmol	Quilonum retard	30.5 mmol
	Trevilor retard	112.5 mg	Trevilor retard	37.5 mg
	Tavor	1.5 mg	Tavor	1.5 mg
			Dipiperon	10 ml
			Chlorhexamed forte	2 applications
			Nortrilen	75 mg
4	Folverlan	5 mg	Folverlan	5 mg
	Tavor	0.75 mg	Tavor	0.25 mg
	Elontril	150 mg	Elontril	300 mg
			Seroquel retard	300 mg
			Januvia	100 mg
			Pantoprazol	40 mg
			Actrapid flex	8 I.E.

5	Mirtazapin	7.5 mg	Mirtazapin	7.5 mg
	Seroxat	40 mg	Seroxat	40 mg
	Tavor	1.5 mg		
6	Quilonum retard	24.4 mmol	Quilonum retard	12.2 mmol
	Mirtazapin	30 mg	Mirtazapin	30 mg
7	Zoloft	150 mg	Zoloft	150 mg
	Pipamperon	40 mg		
	Delix	5 mg	Delix	5 mg
	L-Thyroxin	0.025 mg	L-Thyroxin	0.025 mg
	Quilonum retard	6.1 mmol		
			Doppelherz Multivitamin	1 pellet
			Seroquel	25 mg
			Elontril	300 mg
8	Atosil	100 mg	Atosil	75 mg
	Lyrica	150 mg	Lyrica	150 mg
	Tavor	2 mg		
			Cipralex	15 mg
			Anxut	15 mg
9	Cefuroxim	1000 mg		
	Mirtazapin	7.5 mg		
	Fluoxetin	40 mg		
	Tavor	0.75 mg	Tavor	0.5 mg
	Rupafin	10 mg	Rupafin	10 mg
	L-Thyroxin	0.05 mg	L-Thyroxin	0.05 mg
	Jodid	0.2 mg	Joidid	0.2 mg
	Quilonum retard	18.3 mmol		
			Dipiperon	20 mg
			Movicol	2 sachets
			Trevilor retard	225 mg
			Einmalklistier	2 clysters
10	Sertralin	100 mg	Sertralin	100 mg
	Tavor	0.5 mg		
	Seroquel retard	150 mg	Seroquel retard	150 mg
	L-Thyroxin	0.05 mg	L-Thyroxin	0.075 mg
			Pantoprazol	20 mg
			Colecalciferol	1000 I.E.
11	-		-	

12	L-Thyroxin	0.05 mg	L-Thyroxin	0.05 mg
	Risperdal	3 mg	Risperdal	3 mg
	Tavor	1.5 mg		
	Valsartan	80 mg	Valsartan	80 mg
	Fevarin	125 mg	Fevarin	200 mg
	Stangyl	100 mg	Stangyl	75 mg
13	Amlodipin	5 mg	Amlodipin	5 mg
	Enalapril	10 mg	Enalapril	10 mg
	Esidrix	25 mg	Esidrix	25 mg
	L-Thyroxin	0.125 mg	L-Thyroxin	0.125 mg
	Trevilor retard	150 mg	Trevilor retard	150 mg
	Dipiperon	40 mg		
			Cetirizin	5 mg
14	-		Atorvastatin	10 mg
			Carvedilol	12.5 mg
			Mirtazapin	30 mg

Image reconstruction and processing

PET data were sorted in 30 frames and reconstructed by filtered back projection. All data were corrected for attenuation (via 10 min 68Ge/68Ga transmission scan prior to emissions), random coincidences, and scattering.

Realignment, coregistration, segmentation and normalization of PET and corresponding individual MRI (MPRAGE sequence acquired on a 3T Magnetom Trio, Siemens, Germany) were done with Pmod (version 3.4 and 3.801 PNeuro, PMOD Technologies LLC, Zürich, Switzerland). All data were visually checked for exact segmentation as well as automatic coregistration to corresponding MRI images and were manually corrected if necessary. Data of follow-up PET scans were additionally coregistered to baseline PET to ensure similar orientation of both imaging data sets of one subject. For segmentation, the Hammers N30R83 Maximum Probability Atlas was applied and subsequently dynamic time activity curves (TACs) were generated from PET voxels classified as grey matter with a greater probability of >10%. TACs of both hemispheres were averaged and merged to the regions of interest (ROI) as given in Supplementary Table 2. Correction of blood volume in brain ROIs was done by subtracting 5% of the whole blood activity from the TACs (1).

Results

Supplementary Table 2 General parameters of baseline and follow-up PET scans

**Parameter**	**Baseline**	**Follow-up**	***p* value** (paired t-test)
Start time of emission scan [hh:mm]	12:32 ± 106.88	11:29 ± 86.95	0.072
Injected dose [MBq]	194.31 ± 34.02	215.21 ± 22.69	0.055
Molar activity at time of injection [GBq/µmol]	155.57 ± 88.48	202.45 ± 67.20	0.122
Injected molecular mass [nmol]	1.74 ± 1.12	1.20 ± 0.52	0.123
Mean rate of change of parent compound in plasma [% in min 50-90]	9.73 ± 9.11	10.38 ± 10.93	0.821

Values reported as mean ± standard deviation, n=14

Supplementary Table 3 A_1AR availability before and after ECT treatment

**Region**	**A_1AR availability (V_T min 50-90)**		**%change in V_T across subjects**
	Baseline	Follow-up	
Frontal Cortex	0.73 ± 0.18	0.74 ± 0.22	3.10 ± 22.70
Sensomotor Cortex	0.70 ± 0.18	0.71 ± 0.19	3.40 ± 22.47
Parietal Cortex	0.80 ± 0.21	0.81 ± 0.22	3.53 ± 22.05
Temporal Cortex	0.82 ± 0.19	0.82 ± 0.21	2.12 ± 23.46
Occipital Cortex	0.86 ± 0.20	0.87 ± 0.24	3.14 ± 21.53
Anterior cingulate cortex	0.69 ± 0.19	0.71 ± 0.21	5.93 ± 23.71
Posterior cingulate cortex	0.79 ± 0.20	0.81 ± 0.23	3.64 ± 22.45
Hippocampus	0.75 ± 0.18	0.77 ± 0.20	4.81 ± 24.78
Amygdala	0.72 ± 0.18	0.72 ± 0.18	3.38 ± 23.84
Insula	0.75 ± 0.19	0.74 ± 0.21	2.03 ± 24.50
Cerebellum	0.49 ± 0.10	0.50 ± 0.12	3.53 ± 23.67
Basal Ganglia	0.74 ± 0.19	0.75 ± 0.22	4.51 ± 23.24
Nucleus accumbens	0.59 ± 0.15	0.63 ± 0.16	10.93 ± 27.85
Thalamus	0.75 ± 0.19	0.77 ± 0.20	5.45 ± 23.12
**Across regions**	**0.73 ± 0.18**	**0.74 ± 0.20**	**3.93 ± 22.70**

Values reported as mean ± standard deviation, n=14, A_1AR, A_1 adenosine receptor; V_T, distribution volume in tissue

Supplementary Table 4 Correlation of individual changes of mean A_1AR availability across regions with changes in neuropsychological performance between pre- and post-ECT treatment

Rating	correlation coefficient [r or r_s]	*p* value
HAMD-21	-0.23	0.42
BDI-2	0.01	0.98
GAF	0.23	0.43
MADRS	0.01	0.99
MMSE[1]	0.23	0.43
VLMT	0.30	0.30
WMS (dsf)	0.11	0.71
WMS (dsb)	-0.23	0.43
WMS (stm)	0.16	0.58
WMS (wm)	-0.10	0.73

n=14, [1]correlation coefficient represents Spearman's rho; BDI-2, Beck's Depression Inventory; GAF, Global Assessment of Functioning; HAMD-21, Hamilton Depression Rating Scale; MADRS, Montgomery-Åsberg Depression Rating Scale; MMSE, Mini Mental State Examination; r, Pearson's correlation coefficient; r_s, Spearman's rho; VLMT, Verbal Learning and Memory Test; WMS, Wechsler Memory Scale; dsf, digital span forward; dsb, digital span backward; stm, short-term memory; wm, working memory

Discussion

Potential alteration of A_1AR availability in major depressive disorder (MDD)

Given the A_1AR is not the candidate mediating long-term antidepressant actions of ECT treatment in humans, it remains questionable, if A_1AR expression is altered in severely depressed patients. Ultimately, with the intraindividual design of the present study this question cannot be answered. However, when comparing the current MDD sample consisting of both males and females with previously investigated healthy male controls (2-4), it is striking that the expression patterns of the A_1AR closely resemble each other. The age differences of about 2.5 decades between the study populations however could have led to a decrease of the A_1AR availability in the older sample of the present study (5, 6). But, as such an age-induced reduction of A_1AR availability was dependent on the modelling approach used for quantification of the A_1AR, it remains elusive if a putative reduction might have been canceled out by an increase related to illness activity. In the present sample no correlations of A_1AR availability at baseline and age ranging from 26 to 64 years or clinical depression scores was observed. These findings indicate that most likely A_1AR expression is not altered in severely depressed patients. Moreover, preclinical results already questioned a depression-specific increase in A_1AR availability (see discussion and (7).

Methodological considerations specifically focused on PET imaging and potential influences on outcome parameter distribution volume (V_T)

Test-retest performance of A_1AR quantification with [^{18}F]CPFPX and PET has proven to be stable and reliable (3) and the decomposition of the total variance of changes in A_1AR availability revealed a small within-subject (across regions) and a dominating between-subject share. Medication given to the investigated subjects is not known to have a direct influence on [^{18}F]CPFPX binding to A_1ARs but potential influences, e.g. on metabolism, could not be completely excluded. However, average course of plasma intact ligand during equilibrium analysis for A_1AR quantification revealed no differences between baseline and follow-up PET investigations making it unlikely that metabolism of [^{18}F]CPPFX was systematically influenced by individual medication and its respective changes over time. Moreover, neither subject-specific alterations of plasma intact ligand over time period of equilibrium nor individual changes in parent intact ligand at end of scan time between baseline and follow-up scan correlated with individual variations of A_1AR availability. Alternative approaches of A_1AR quantification independent of an equilibrium of radioligand plasma and tissue concentration, namely analysis via Logan Plot (8) and non-invasive Logan Plot (9) with the cerebellum as reference region, lead to comparable results. Even more, uptake of radioligand into the brain in terms of an area-under-the-curve analysis did not indicate any dependency on unbound ligand in plasma (which might vary due to plasma-protein binding of drugs and subsequent competition with the radioligand) further emphasizing that the outcome parameter V_T is not affected by individual medication in the current sample.

Reference List

1. Leenders KL, Perani D, Lammertsma AA, Heather JD, Buckingham P, Healy MJ, et al. Cerebral blood flow, blood volume and oxygen utilization. Normal values and effect of age. Brain. 1990;113 (Pt 1):27-47.
2. Elmenhorst D, Elmenhorst EM, Hennecke E, Kroll T, Matusch A, Aeschbach D, et al. Recovery sleep after extended wakefulness restores elevated A(1) adenosine receptor availability in the human brain. Proc Natl Acad Sci U S A. 2017;114(16):4243-8.
3. Elmenhorst D, Meyer PT, Matusch A, Winz OH, Zilles K, Bauer A. Test-retest stability of cerebral A1 adenosine receptor quantification using [18F]CPFPX and PET. Eur J Nucl Med Mol Imaging. 2007;34(7):1061-70.
4. Elmenhorst D, Meyer PT, Winz OH, Matusch A, Ermert J, Coenen HH, et al. Sleep deprivation increases A1 adenosine receptor binding in the human brain: a positron emission tomography study. J Neurosci. 2007;27(9):2410-5.
5. Meyer PT, Elmenhorst D, Boy C, Winz O, Matusch A, Zilles K, et al. Effect of aging on cerebral A1 adenosine receptors: A [18F]CPFPX PET study in humans. Neurobiol Aging. 2007;28(12):1914-24.
6. Mishina M, Kimura Y, Sakata M, Ishii K, Oda K, Toyohara J, et al. Age-Related Decrease in Male Extra-Striatal Adenosine A(1) Receptors Measured Using(11)C-MPDX PET. Front Pharmacol. 2017;8:903.
7. Crema LM, Pettenuzzo LF, Schlabitz M, Diehl L, Hoppe J, Mestriner R, et al. The effect of unpredictable chronic mild stress on depressive-like behavior and on hippocampal A1 and striatal A2A adenosine receptors. Physiol Behav. 2013;109:1-7.
8. Logan J, Fowler JS, Volkow ND, Wolf AP, Dewey SL, Schlyer DJ, et al. Graphical analysis of reversible radioligand binding from time-activity measurements applied to [N-11C-methyl]-(-)-cocaine PET studies in human subjects. J Cereb Blood Flow Metab. 1990;10(5):740-7.
9. Logan J, Fowler JS, Volkow ND, Wang GJ, Ding YS, Alexoff DL. Distribution volume ratios without blood sampling from graphical analysis of PET data. J Cereb Blood Flow Metab. 1996;16(5):834-40.
